# The role of comorbid hypertriglyceridemia and abdominal obesity in the severity of acute pancreatitis: a retrospective study

**DOI:** 10.1186/s12944-021-01597-4

**Published:** 2021-11-27

**Authors:** Xiaoxi Yang, Jiajun He, Shuli Ma, Tingting Wang, Quping Zhu, Fei Cao, Yuanhao Li, Chuting Yang, Chaowu Chen, Guotao Lu, Lianghao Hu, Jun Liu, Weiwei Chen

**Affiliations:** 1 Department of Gastroenterology, Clinical Medical College, Yangzhou University, No 98, Nantong West Rd, Yangzhou, Jiangsu Province, 225000 China; 2 School of Nursing, Yangzhou University, Yangzhou, China; 3 Department of Gastroenterology, Affiliated Hospital of Nantong University, Nantong, China; 4 Department of Gastroenterology, Affiliated Hospital of Yangzhou University, Yangzhou, China; 5 Department of Gastroenterology, Changhai Hospital, Second Military Medical University, Shanghai, China

**Keywords:** Hypertriglyceridemia, Abdominal obesity, Acute pancreatitis

## Abstract

**Background:**

The effect of comorbid hypertriglyceridemia (HTG) and abdominal obesity (AO) on acute pancreatitis (AP) remains unclear. The aim of this study was to explore the effect of comorbid HTG and AO and discuss which is the dominant disorder.

**Methods:**

In this study, 1219 AP patients who presented with HTG or AO were stratified into four groups: non-HTG + non-AO, HTG + non-AO, non-HTG + AO, and HTG + AO.

**Results:**

The 328 patients with comorbid HTG + AO were much younger (42.29 ± 11.77), mainly male (79.57%), and had higher TG levels, larger waist circumferences, and more past medical histories than the patients in the other three non-comorbid groups (*P* < 0.001). The comorbidity group developed more incidences of persistent organ failure and local complications (*P* < 0.05). Multivariate logistic regression analysis showed that AO (OR = 3.205, 95% CI = 1.570–6.544), mild HTG (OR = 2.746, 95% CI = 1.125–6.701), and moderate to very severe HTG (OR = 3.649, 95% CI = 1.403–9.493) were independent risk factors for persistent respiratory failure (*P* < 0.05). Age > 60 years (OR = 1.326, 95% CI = 1.047–1.679), AO (OR = 1.701, 95% CI = 1.308–2.212), diabetes mellitus (OR = 1.551, 95% CI = 1.063–2.261), mild HTG (OR = 1.549, 95% CI = 1.137–2.112), and moderate to very severe HTG (OR = 2.810, 95% CI = 1.926–4.100) were independent risk factors associated with local complications (*P* < 0.05). Moreover, HTG seemed to be more dangerous than AO. The higher the serum TG level was, the greater the likelihood of persistent respiratory failure and local complications.

**Conclusions:**

Comorbid HTG and AO will aggravate the severity and increase the incidence of local complications of AP. HTG may play a dominant role of risk in the condition of comorbidity.

**Chinese clinical trial registry:**

ChiCTR2100049566. Registered on 3^rd^ August, 2021. Retrospectively registered, https://www.chictr.org.cn/edit.aspx?pid=127374&htm=4.

## Introduction

Acute pancreatitis is a common inflammatory disease in the digestive system [1]. The global incidence of AP is approximately 4.9–80/100,000, and it has been increasing worldwide [2]. Generally, mild acute pancreatitis (MAP) is characterized by a short duration and good prognosis. However, once severe acute pancreatitis (SAP) develops, the mortality rate is as high as 30–50% [3, 4]. Therefore, early and accurate identification of high-risk AP patients is of great significance to reduce mortality and improve prognosis.

Recently, studies have indicated that abnormal lipid metabolism is closely associated with AP [5, 6]. As a key lipometabolic disorder, hypertriglyceridemia (HTG) has been recognized to be a cause of AP and an aggravating factor in AP progression [7]. Clinical studies have shown that the level of serum triglycerides (TGs) is an independently risk factors for persistent organ failure (POF) in AP patients [1]. In addition, obesity has been proven to be an independent risk factor for AP [8, 9], which aggravates the severity, prolongs hospital stay, and increases mortality [10, 11], especially for patients with abdominal obesity (AO) caused by excessive visceral fat [12]. However, few studies have researched the effect of comorbid HTG and AO on the severity and local complications of AP. Therefore, the objective of this study is to explore the influence of these comorbidities and discuss which is the dominant disorder.

## Materials and methods

### Study population

**Fig. 1:**
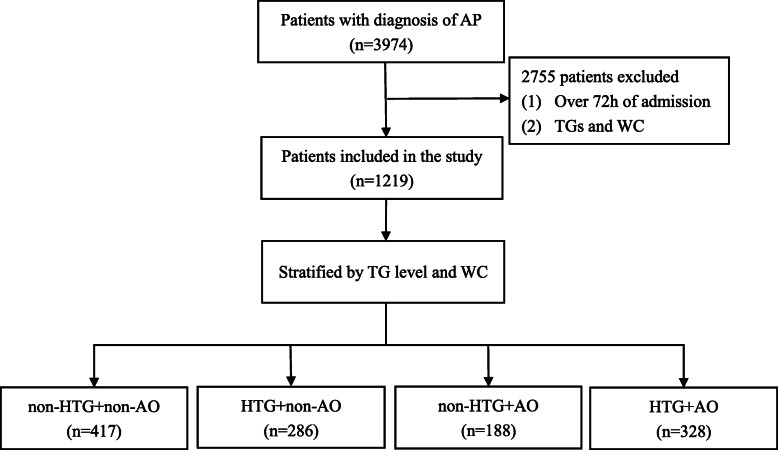
The flow chart of patients’ selection process. AP, acute pancreatitis; WC, waist circumference; HTG, hypertriglyceridaemia; AO, abdominal obesity

This retrospective observational study was carried out in the Department of Gastroenterology and was approved by the ethics committee of the hospital on August 4^th^, 2020 (No. 2020ky-047). Written informed consent was obtained from each patient. The research protocol was complied with the ethical guidelines of the 1975 Declaration of Helsinki. It was registered in the Chinese Clinical Trial Registry (Registration number: ChiCTR2100049566). The data were obtained from hospital information system (HIS) between January 1^st^, 2016, and December 31^st^, 2020. Patients with AP aged 18 to 80 years old were included in this study. They were hospitalized within 7 days of onset, with TG levels and waist circumference (WC) available in the first 3 days. The diagnosis and classification of AP was made according to the revision of the Atlanta classification [4]. Exclusion criteria were a. serious comorbidities at admission, such as chronic renal failure, b. any history of cancer, c. participation in other clinical trials that might interfere with this study, or d. pregnancy or parturient women. The screening process for patients is shown in Fig. 1.

### Definition and classification of HTG and AO

According to the Endocrine Society Clinical Practice Guidelines [13], HTG level was determined above 1.7 mmol/L [13–16]. In this study, HTG was divided into two groups according to TG levels: mild (1.7 mmol/L ≤ TG < 5.65 mmol/L) and moderate to severe (TG ≥ 5.65 mmol/L).

AO was defined by WC, which was estimated by abdominal CT imaging. The horizontal and vertical axes of the navel on abdominal CT were measured. Then, the WC was calculated using the standard ellipse formula (WC = the elliptical coefficient × (short axis + long axis) ÷2)) [17]. Based on the Chinese criteria of AO for adults, men with WC ≥ 90 cm or women with WC ≥ 85 cm are considered to have AO.

In this study, four groups were established based on the above criteria: Group 1 (non-HTG + non-AO): TG < 1.7 mmol/L, WC < 90 cm (for men) or WC < 85 cm (for women), Group 2 (HTG + non-AO): TG ≥ 1.7 mmol/L, WC < 90 cm (for men) or WC < 85 cm (for women), Group 3 (non-HTG + AO): TG < 1.7 mmol/L, WC ≥ 90 cm (for men) or WC ≥ 85 cm (for women), and Group 4 (HTG + AO): TG ≥ 1.7 mmol/L, WC ≥ 90 cm (for men) or WC ≥ 85 cm (for women).

### Data collection

Data analysed in this study included demographic data such as sex, age, WC, aetiology, episodes of AP, past medical history, clinical features and vital signs, laboratory indices, and image data. All data were obtained from the HIS and recorded in the unified case report form. Quality control was carried out in data collection to ensure accuracy.

### Statistical analysis

Statistical analysis was performed with IBM Statistical Packages for the Social Sciences (SPSS®) 26.0. Normally distributed data are expressed as the mean ± standard deviation, and nonnormally distributed data are expressed as the median and extreme ranges. Categorical data were analysed by χ^2^ test or 2-tailed Fisher’s exact test. One-way ANOVA was applied between the four groups. Mann–Whitney U tests were used for pairwise comparisons among the groups. Multivariate binary logistic regressions (Wald’s test) were performed to evaluate the risk factors for persistent respiratory failure and local complications. Statistical significance was determined with *P* value of < 0.05.

## Results

### Patients’ demographics and clinical characteristics

There were 1219 AP patients included in this study. The 328 patients with comorbid HTG and AO were much younger (42.29 ± 11.77) and mainly male (79.57%) compared to patients in the other three groups. The TG levels in the two HTG groups were much higher than those in the two non-HTG groups, whereas the WC was significantly higher in the obesity groups than in the non-AO groups. In terms of aetiology, biliary was the main cause in the non-HTG + non-AO and non-HTG + AO group (65.71 and 67.55%, respectively), whereas HTG was the major cause in the HTG + non-AO and HTG + AO groups (54.55 and 59.76%, respectively). In past medical history, recurrence ≥2 times and proportions of diabetes mellitus, alcoholism, and smokers were much higher in the comorbid group than in the other three groups, followed by the HTG + non-AO group. However, there were no significant differences between the non-HTG + non-AO group and the non-HTG + AO group. No significant difference was found in hospital stay (*P* = 0.690). The patients’ demographics were shown in Table 1.

**Table 1:** Patients’ demographics and clinical characteristics in the four groups of AP

Variables	non-HTG + non-AO (***n*** = 417)	HTG + non-AO (***n*** = 286)	non-HTG + AO (***n*** = 188)	HTG + AO (***n*** = 328)	***P***
Sex (n, %)					< 0.001
Male	220 (52.76)	182 (63.64) ^***a***^	80 (42.55) ^***b***^	261 (79.57) ^***a b c***^	
Female	197 (47.24)	104 (36.36) ^***a***^	108 (57.45) ^***a b***^	67 (20.43) ^***a b c***^	
Age (Years) (mean ± SD)	60.26 ± 16.08	48.65 ± 12.06 ^***a***^	60.00 ± 16.67 ^***b***^	42.29 ± 11.77 ^***a b c***^	< 0.001
TG (mmol/L) [median (range)]	0.82 (0.15–1.69)	4.69 (1.71–116.38) ^***a***^	1.06 (0.28–1.68) ^***a b***^	7.31 (1.72–85.60) ^***a c***^	< 0.001
WC (cm) (mean ± SD)	78.86 ± 6.29	81.83 ± 6.31 ^***a***^	93.14 ± 5.98 ^***a b***^	96.59 ± 6.70 ^***a b c***^	< 0.001
Aetiologies (n, %)					< 0.001^*^
Biliary	274 (65.71)	54 (18.88) ^***a***^	127 (67.55) ^***b***^	59 (17.99) ^***a c***^	
Hypertriglyceridemia	0 (0.00)	156 (54.55) ^***a***^	0 (0.00) ^***b***^	196 (59.76) ^***a c***^	
Alcoholic	12 (2.88)	17 (5.94)	5 (2.66)	15 (4.57)	
ERCP	2 (0.48)	0 (0.00)	0 (0.00)	1 (0.30)	
Idiopathic	99 (23.74)	49 (17.13)	48 (25.53)	46 (14.02) ^***a c***^	
Mixedness	1 (0.24)	4 (1.40)	1 (0.53)	6 (1.83)	
Others	29 (6.95)	6 (2.10) ^***a***^	7 (3.72)	5 (1.52) ^***a***^	
Recurrence≥2 (Times) (n, %)	35 (8.39)	33 (11.54)	10 (5.32)	54 (16.46) ^***a c***^	< 0.001
Diabetes mellitus (n, %)	44 (10.55)	68 (23.78) ^***a***^	26 (13.83) ^***b***^	84 (25.61) ^***a c***^	< 0.001
Alcoholism (n, %)	54 (12.95)	67 (23.43) ^***a***^	21 (11.17) ^***b***^	92 (28.05) ^***a c***^	< 0.001
Smoker (n, %)	51 (12.23)	54 (18.88)	16 (8.51) ^***b***^	91 (27.74) ^***a c***^	< 0.001
Hospital stay (Day) (mean ± SD)	9.41 ± 4.61	9.21 ± 4.81	9.61 ± 4.76	9.66 ± 5.46	0.690

TG: serum triglyceride; WC: waist circumference; ERCP: endoscopic retrograde cholangiopancreatography; ^*a*^ *P* < 0.05, compared with the non-HTG + non-AO group; ^*b*^ *P* < 0.05, compared with the HTG + non-AO group; ^*c*^ *P* < 0.05, compared with the non-HTG + AO group; * Fisher’s exact test

### Clinical outcomes

The 1219 AP patients included 831 (68.17%) patients with moderately severe acute pancreatitis (MSAP), 54 (4.43%) patients with SAP with POF, 33 (2.71%) patients with transient organ failure (TOF), and 205 (16.82%) patients with systemic inflammatory response syndrome (SIRS). A significant difference was found in the severity of AP among the four groups (*P* < 0.001). There was 75.30% MSAP and 7.32% SAP in the HTG + AO comorbidity group, followed by the HTG + non-AO group with 75.52% MSAP and 2.45% SAP, the non-HTG + AO group with 65.96% MSAP and 4.79% SAP, and the non-HTG + non-AO group with 58.51% MSAP and 3.36% SAP. There was no significant difference in TOP. However, a significant difference was found in POF. The incidence of persistent respiratory failure was significantly higher in the comorbid group than in the other three groups (*P* = 0.002). Similarly, the incidence of SIRS (25.91%) was the highest and significantly different in the comorbid group (*P* < 0.001) (Table 2).

**Table 2:** Comparison of the severity in the four group in patients with AP

Variables	non-HTG + non-AO (***n*** = 417)	HTG + non-AO (***n*** = 286)	non-HTG + AO (***n*** = 188)	HTG + AO (***n*** = 328)	***P***
Atlanta classification (n, %)				< 0.001	
MAP	159 (38.13)	63 (22.03) ^***a***^	55 (29.25)	57 (17.38) ^***a c***^	
MSAP	244 (58.51)	216 (75.52) ^***a***^	124 (65.96)	247 (75.30) ^***a c***^	
SAP	14 (3.36)	7 (2.45)	9 (4.79)	24 (7.32) ^***a b***^	
OF (n, %)	24 (5.76)	15 (5.24)	14 (7.45)	32 (9.76)	0.100
POF (n, %)	14 (3.36)	7 (2.45)	9 (4.79)	24 (7.32) ^***a b***^	0.016
Persistent respiratory failure	8 (1.92)	6 (2.10)	7 (3.72)	22 (6.71) ^***a b***^	0.002
Persistent heart failure	2 (0.48)	0 (0.00)	1 (0.53)	1 (0.30)	0.277^*^
Persistent renal failure	5 (1.20)	2 (0.70)	4 (2.13)	3 (0.91)	0.564^*^
TOF (n, %)	12 (2.88)	8 (2.80)	5 (2.66)	8 (2.44)	0.986
SIRS (n, %)	49 (11.75)	48 (16.78)	23 (12.23)	85 (25.91) ^*a b c*^	< 0.001

OF: organ failure; POF: persistence organ failure; TOF: transient organ failure; SIRS: systemic inflammatory response syndrome; ^*a*^ *P* < 0.05, compared with the non-HTG + non-AO group; ^*b*^ *P* < 0.05, compared with the HTG + non-AO group; ^*c*^ *P* < 0.05, compared with the non-HTG + AO group; * Fisher’s exact test

Additionally, 885 (72.60%) patients developed local complications, and 842 (69.07%) had acute peripancreatic fluid collection (APFC), 55 (4.51%) suffered from acute necrotic collection (ANC), 31 (2.54%) developed pancreatic pseudocysts (PPCs), 4 (0.33%) had walled-off necrosis (WON), and 1 (0.08%) endured infectious pancreatic necrosis (IPN). Significant differences were found among the four groups for local complications (*P* < 0.001). In particular, the incidences of APFC and ANC were higher in the comorbid group than in the other three groups (*P* < 0.05). It was high up to 79.88% of APFC and 8.23% of ANC in the HTG + AO comorbidity group, followed by the HTG + non-AO group with 73.78% of APFC and 3.85% of ANC, the non-HTG + AO group with 69.68% of APFC and 3.72% of ANC, and the non-HTG + non-AO group with 57.07% of APFC and 2.40% of ANC. No significant differences were identified in PPC, WON, or IPN among the four groups. The MCTSI score was significantly different among the four groups (*P* < 0.001). However, there was no difference between the comorbid group and the HTG + non-AO group (Table 3).

**Table 3:** Comparison of local complications in the four groups in patients with AP

Variables	non-HTG + non-AO	HTG + non-AO	non-HTG + AO	HTG + AO	***P***
	(***n*** = 417)	(***n*** = 286)	(***n*** = 188)	(***n*** = 328)	
Local Complications (n, %)	259 (62.11)	218 (76.22) ^***a***^	139 (73.94)	269 (90.24) ^***a c***^	< 0.001
APFC (n, %)	238 (57.07)	211 (73.78) ^***a***^	131 (69.68)	262 (79.88) ^***a c***^	< 0.001
ANC (n, %)	10 (2.40)	11 (3.85)	7 (3.72)	27 (8.23) ^***a***^	0.001
PPC (n, %)	7 (1.68)	6 (2.10)	4 (2.13)	14 (4.27)	0.135
WON (n, %)	0 (0.00)	1 (0.35)	0 (0.00)	3 (0.91)	0.098^*^
IPN (n, %)	0 (0.00)	1 (0.35)	0 (0.00)	0 (0.00)	0.407^*^
MCTSI (mean ± SD)	4.09 ± 2.25	4.94 ± 1.75 ^***a***^	4.29 ± 2.07 ^***b***^	4.74 ± 1.86 ^***a c***^	< 0.001

APFC: acute peripancreatic fluid collection; ANC: acute necrotic collection; PPC: pancreatic pseudocyst; WON: walled-off necrosis; IPN: infectious pancreatic necrosis; OF: organ failure; POF: persistent organ failure; TOF: transient organ failure; SIRS: systemic inflammatory response syndrome; MCTSI: modification of CT severity index; ^*a*^ *P* < 0.05, compared with the non-HTG + non-AO group; ^*b*^ *P* < 0.05, compared with the HTG + non-AO group; ^*c*^ *P* < 0.05, compared with the non-HTG + AO group; * Fisher’s exact test

### Logistic regression analysis

Logistic regression analysis was applied to explore the risk factors for persistent respiratory failure and local complications. The univariate analysis indicated that AO, diabetes mellitus, and HTG were risk factors for persistent respiratory failure (*P* < 0.05, Table 4). Furthermore, sex (male), age (> 60 years), AO, diabetes mellitus, alcoholism, smoking, and HTG were also risk factors for local complications, with significant differences (*P* < 0.05, Table 5).

**Table 4:** Univariate Logistic regression analysis of persistent respiratory failure in AP patients

Variable	B (S.E)	Adjusted OR	95% CI	***P***
Male	0.363	1.437	0.776–2.663	0.249
Age > 60 years	0.254	1.290	0.666–2.498	0.451
AO	1.272	3.567	1.808–7.037	< 0.001
Diabetes mellitus	0.731	2.076	1.061–4.062	0.033
Recurrence ≥2 times	0.148	0.863	0.303–2.457	0.782
Alcoholism	0.366	0.694	0.289–1.666	0.413
Smoking	0.411	1.508	0.730–3.118	0.267
HTG	0.420	1.522	1.062–2.181	0.022
Mild	0.546	1.726	0.788–3.779	0.172
Moderate to severe	0.844	2.325	1.119–4.829	0.024

**Table 5:** Univariate Logistic regression analysis of local complications in AP patients

Variable	B (S.E)	Unadjusted OR	95% CI	***P***
Male	1.075	2.931	2.485–3.457	< 0.001
Age > 60 years	0.818	2.266	1.834–2.799	< 0.001
AO	1.338	3.813	3.082–4.718	< 0.001
Diabetes mellitus	1.455	4.286	3.063–5.996	< 0.001
Recurrence ≥2 times	0.094	1.099	0.728–1.659	0.654
Alcoholism	0.556	1.744	1.225–2.482	0.002
Smoking	0.682	1.978	1.354–2.892	< 0.001
HTG	0.573	1.774	1.649–1.908	< 0.001
Mild	1.039	2.835	2.189–3.645	< 0.001
Moderate to severe	1.736	5.674	4.147–7.762	< 0.001

To avoid confounding factors, stepwise multivariate logistic regression analyses of risk factors for persistent respiratory failure and local complications were performed. The results are summarized in Table 6 and Table 7. AO (OR = 3.205, 95% CI = 1.570–6.544), mild HTG (OR = 2.746, 95% CI = 1.125–6.701), and moderate to very severe HTG (OR = 3.649, 95% CI = 1.403–9.493) were indicated as independent risk factors for persistent respiratory failure (*P* < 0.05) (Table 6). Age > 60 years (OR = 1.326, 95% CI = 1.047–1.679), AO (OR = 1.701, 95% CI = 1.308–2.212), diabetes mellitus (OR = 1.551, 95% CI = 1.063–2.261), mild HTG (OR = 1.549, 95% CI = 1.137–2.112), and moderate to very severe HTG (OR = 2.810, 95% CI = 1.926–4.100) were independent risk factors associated with local complications (*P* < 0.05) (Table 7). Moreover, HTG seemed to be more dangerous than AO (*P* < 0.05). The higher the serum TG level was, the greater the likelihood of persistent respiratory failure and local complications.

**Table 6:** Multiple Logistic regression analysis of persistent respiratory failure in AP patients

Variable	B (S.E)	Unadjusted OR	95% CI	***P***
AO	1.165	3.205	1.570–6.544	0.001
Diabetes mellitus	0.462	1.587	0.779–3.233	0.203
HTG				
Mild	1.010	2.746	1.125–6.701	0.026
Moderate to severe	1.295	3.649	1.403–9.493	0.008

**Table 7:** Multiple Logistic regression analysis of local complications in AP patients

Variable	B (S.E)	Unadjusted OR	95% CI	***P***
Male	0.166	1.180	0.925–1.507	0.183
Age > 60 years	0.282	1.326	1.047–1.679	0.019
AO	0.531	1.701	1.308–2.212	< 0.001
Diabetes mellitus	0.439	1.551	1.063–2.261	0.023
Alcoholism	0.216	1.241	0.805–1.915	0.329
Smoking	0.444	1.559	0.984–2.469	0.059
HTG				
Mild	0.438	1.549	1.137–2.112	0.006
Moderate to severe	1.033	2.810	1.926–4.100	< 0.001

## Discussion

With the improvement in living standards and the adjustment of dietary structure, the incidence of hypertriglyceridemic AP (HTG-AP) has increased significantly, and HTG has become the second major cause of AP in China [18]. Abdominal adiposity has an adverse impact on AP [19] and shows an obvious trend of increasing severity. Although HTG and AO have been associated with the severity of AP in previous studies, few studies have examined the effect of comorbid HTG and AO on AP. This study first proposed an association between comorbid HTG and AO and the severity of AP. The study demonstrated that HTG and AO were independent risk factors associated with persistent respiratory failure and local complications in AP patients. Moreover, the risk of HTG was higher than that of AO. The higher the serum TG level was, the greater the likelihood of persistent respiratory failure and local complications, which suggested that HTG may play a dominant role in AP risk.

This study found that 42.17% of patients exhibited biliary AP, followed by 28.88% with HTG-AP, indicating that HTG is the second major cause of AP in the centre. Patients with comorbid HTG and AO were significantly younger and were mainly male, which was consistent with a previous study [20]. This study also showed that recurrence, metabolic comorbidity of diabetes, and a lifestyle of drinking and smoking were much higher in the comorbid group. This pattern may be related to long-term drinking, a high-fat diet, a lack of exercise and other poor living habits among young men [21], thus leading to abnormal fat metabolism and other phenomena.

Additionally, the results showed that patients with comorbid HTG and AO had higher incidences of SAP, persistent organ failure, SIRS, and local complications, especially APFC and ANC. Study demonstrated that elevated TG may lead to respiratory insufficiency by affecting pulmonary gas exchange [22]. The results of the study were consistent with those of Chen et al. [23], who pointed out that HTG and obesity are definitely risk factors for the local complications of AP. Patel et al. also showed that mild AP develops into SAP in obese mice with increased MOF [24]. In addition, this study also found that HTG was a more dangerous pathogenic factor than AO. The higher the serum TG level was, the greater the likelihood of persistent respiratory failure and local complications, which suggested that HTG may play a dominant role of risk in the condition of comorbidity. This finding has never been reported before.

The mechanisms by which HTG and AO aggravate AP may include adipokines, cytokines, unsaturated fatty acid (UFA)-mediated lipotoxicity, and oxidative stress. Previous studies reported that the severity of AP is related to an increase in visceral fat [25, 26], which accounts for 1–10% of body weight [27]. TG oversteps more than 80% of adipocyte mass [28], which is hydrolysed into free fatty acid (FFA) and results in lipotoxicity of acinar cells. Insoluble TG may result in pancreatic microthrombi, ischaemia and infarction. Exocrine pancreatic acinar cells are rich in lipase, which is responsible for hydrolysing TG into UFAs [29]. UFAs may worsen inflammatory conditions and release intracellular calcium, thereby causing acinar necrosis by decreasing ATP levels [8]. It can also aggregate into micelles and cause pancreatic ischaemia [30], which activates lysosomal pepsine-B and trypsinogen, resulting in acidosis, pancreatic self-digestion, and injury [31]. Excessive UFAs induce apoptosis of alveolar epithelia and renal tubular injury [1, 32]. In addition, FFA directly influence on pancreatic acinus [33], stimulating the secretion of TNF-α, IL-1β, MCP-1, IL-18, and others [34]. In addition, high-fat diet-induced hyperlipidaemia is related to oxidative stress and ROS, which are considered key factors causing tissue damage and the inflammatory response in AP [35]. All the above results indicated that comorbid HTG and AO would aggravate the severity of AP. AO may act through HTG. Thus, HTG may play a primary role of risk in comorbidities.

### Comparisons with other studies and what the current work adds to existing knowledge

Ding et al. [36] pointed out that the waist phenotype of HTG was related to the severity of AP. Kiss et al. showed in a meta-analysis that the presence of HTG (even at mild levels) exacerbates the condition of AP [37]. Compared with previous studies, this study proved the influence of comorbid HTG and AO on the progression of AP. The complications of HTG and AO further aggravated the severity and increased the incidence of local complications of AP, especially persistent respiratory failure, APFC and ANC. Interestingly, there is a preliminary indication that HTG is more dangerous than AO in the course of AP.

### Strengths and limitations

As described above, this study explored the influence of comorbid HTG and AO on the severity of AP and found that HTG was more dangerous than AO. However, there were also several limitations in the study. First, as TG levels decreased rapidly with fasting, the TG levels within the first three days after admission were underestimated. Therefore, unintended bias may have occurred. Second, abdominal CT was not the most appropriate way to measure WC, but all subjects included adopted the same measurement method, which may avoid bias to some extent.

## Conclusions

In conclusion, comorbid HTG and AO will aggravate the severity and increase the incidence of local complications of AP, especially persistent respiratory failure, APFC and ANC. HTG may play a dominant role of risk in comorbidities. More attention should be given to AP patients with comorbid HTG and AO in the future. In the early stage of AP, medical staff should pay close attention to such patients and give effective treatment in time to prevent complications. Additionally, in the recovery period, health education, such as diet management and AO slimming, should be carried out, and TG levels should be monitored regularly to reduce the recurrence of AP.

## Data Availability

All data are contained in the article. The raw data will be shared upon request: Contact corresponding author.

## Abbreviations

AP: Acute pancreatitis
APFC: Acute peripancreatic fluid collection
ANC: Acute necrotic collection
CRF: Case report form
ERCP: Endoscopic retrograde cholangiopancreatography
FFA: Free fatty acid
HTG: Hypertriglyceridemia
HTG-AP: Hypertriglyceridemic acute pancreatitis
IPN: Infectious pancreatic necrosis
MAP: Mild acute pancreatitis
MCTSI: Modification of CT severity index
OR: Odds ratio
OF: Organ failure
POF: Persistence organ failure
PPCs: Pancreatic pseudocysts
SAP: Severe acute pancreatitis
SIRS: Systemic inflammatory response syndrome
TG: Serum triglyceride
TOF: Transient organ failure
UFAs: Unsaturated fatty acids
WC: Waist circumference
WON: Walled-off necrosis
